# Effectiveness of community organizing interventions on social activities among older residents in Japan: A JAGES quasi-experimental study

**DOI:** 10.1016/j.socscimed.2019.112527

**Published:** 2019-11

**Authors:** Maho Haseda, Daisuke Takagi, Katsunori Kondo, Naoki Kondo

**Affiliations:** a Department of Health Education and Health Sociology, School of Public Health, The University of Tokyo, Tokyo, Japan; b Department of Health and Social Behavior, School of Public Health, The University of Tokyo, Tokyo, Japan; c Center for Preventive Medical Sciences, Chiba University, Chiba, Japan; d Center for Gerontology and Social Science, National Center for Geriatrics and Gerontology, Aichi, Japan

**Keywords:** Community organizing, Quasi-experimental study, Aged, Japan, Group participation

## Abstract

Social activities in the community help older adults maintain functional ability. Community organizing, based on the assessment of health risks, community assets, and fostering intersectoral organizational partnerships, could increase participation opportunities. Supporting municipality staff members in building their capacity to take those actions might benefit them. Nevertheless, the effectiveness of such support remains unclear. This real-world-setting study evaluated the effectiveness of providing support for municipality health sectors in relation to older residents’ social activities.

Based on the Japan Gerontological Evaluation Study (JAGES), a nationwide study of community-dwelling older adults, from 2013 to 2016 researchers collaborated with health sector staff members in 13 participating municipalities (intervention group) in utilizing the JAGES-based community assessment data and building organizational partnerships. The remaining 12 municipalities (control) obtained the data only. We analyzed the longitudinal data of 47,106 older residents, performing a difference-in-differences (DID) analysis, weighted by the inverse of propensity to be selected for the intervention group, allowing for a multilevel (municipality/individual) data structure.

In the intervention group, the estimated group participation prevalence in men increased by 10.4 percentage points from 47.5% to 57.9%, while in the control group, participation increased by 7.9 percentage points from 47.2% to 55.0% (DID estimated = 0.025, P = 0.011). No statistically significant difference between the two groups was observed among women (P = 0.131).

Support for community organizing may improve group participation among older male residents. The community-attributable impact could be large, given that the intervention has the potential to work for all older residents in the municipality.

## Highlights

• Older men participated more where researchers and health sector collaborated well.
• The effects were specifically strong for participation in leisure activity groups.
• Community organizing might encourage older men to engage in social activities.

## 1 Introduction

The growing number of disabled older adults is a major public health issue in an aging world (World Health Organization, 2017). The World Health Organization is promoting a new agenda, Healthy Ageing, and emphasizes creating communities that provide opportunities not only for preventive care services but also social activities, in which older adults can maintain their physical, mental, cognitive, and social health. Studies show that individual psychosocial conditions, including social relationships and group participation in the community, benefit older adults’ functional ability (Holt-Lunstad et al., 2010; Kanamori et al., 2014; Hikichi et al., 2015). To foster the opportunities for such participation, community-level collaborative actions based on partnerships with multiple public and private sectors, and with civic organizations, are required (World Health Organization, 2017).

In community-related interventions, strong top-down regulations and political reforms could be powerful measures (Naidoo and Wills, 2016), but caution is needed as this also risks ignoring residents’ needs and the resources needed for effective interventions. Community empowerment and an organizing approach is a useful alternative in meeting residential intervention needs, creating networks and dialogues with various stakeholders to provide support to the community. Minkler defined community organizing as “the process by which community groups are helped to identify common problems or change targets, mobilize resources, and develop and implement strategies to reach their collective goals” (Minkler, 2012).

To carry out community organizing, community assessment and the ability to build intersectoral partnerships are crucial skills for the practitioners in local public health sectors (Ståhl et al., 2006, Public Health Agency of Canada, 2007). As there is evidence that local staff members often lack sufficient skills and resources for using epidemiological data and collaborating with other sectors or organizations, supporting frameworks for the staff members of local health sectors are required (Ollila, 2011; Larsen et al., 2014). Recent studies have suggested that broad cooperation among indigenous social agents and grassroots organizations could be associated with the enhancement of residents’ subjective health status, improved control of chronic diseases, and self-efficacy among residents (Jung and Viswanath, 2013; Pibernik-Okanovic et al., 2004; DeCoster and George, 2005).

Such community-related interventions through collaboration between local health sector staff and researchers would be beneficial for older people who often spend much time in their residential community. However, to the best of our knowledge, there are few studies which focus on the effectiveness of such community organizing interventions aimed at older adults. Moreover, according to Shearer et al., it is difficult to draw conclusions about the effectiveness of those community organizing interventions because of a lack of consistency across studies regarding measurements, intervention strategy, and study sizes. In particular, many studies focus on relatively small communities (Shearer et al., 2012).

Thus, the aim of this study was to elucidate the effectiveness of community-organizing interventions on older adults' participation in social activities. The study was in a real-world setting, used a longitudinal quasi-experimental design, had a very large sample size, and involved multiple areas in Japan, the country with the world's most aged population.

## 2 Methods

### 2.1 Data

The Japan Gerontological Evaluation Study (JAGES) is a large-scale, population-based collaborative research study between researchers and municipalities, aimed at exploring the social determinants of longevity in older adults. We used JAGES panel data from the 2013 and 2016 datasets. The 2013 baseline JAGES survey was conducted between October and December 2013, while the follow-up survey was conducted between October and December 2016. The mean follow-up period was 1092 days across 25 municipalities in 11 out of 47 prefectures in Japan. In the 2013 survey, we mailed self-administered questionnaires to community-dwelling individuals aged 65 years and older who were functionally independent in their daily living. In the survey we used a multistage random sampling method in 12 large municipalities based on the official residential registers and mailed all eligible residents living in 13 small municipalities (N = 162,496). The baseline sample in 2013 comprised 114,655 participants (response rate: 70.6%). Among them, we contacted 85,422 participants in the 2016 survey after the exclusion of participants who had passed away, developed functional disability (i.e., received benefits from public long-term care insurance [LTCI]), or had moved to another municipality during the follow-up period. Of the participants (N = 64,766), 75.8% completed the follow-up questionnaire in 2016. Participants without valid responses to the questions on group participation and frequency of going out were excluded from the analysis.

### 2.2 Intervention

The JAGES research team has visualized and diagnosed community-related health issues, using a community benchmarking framework called the JAGES Health Equity Assessment and Response Tool (JAGES-HEART) (Ojima and JAGES project, 2014). After the survey, researchers aggregate individual questionnaire responses by municipality and small areas within municipalities and create community diagnosis forms. The forms provided between- and within-municipality comparisons on regional health statuses and daily life activities, including community group participation, the frequency of going out, and other factors we have reported on in this paper. The researchers have provided these community assessment tools to the local health sector in every municipality and have held seminars (group sessions for municipality officials) to assess and utilize the results for community health promotion.

Among the 25 municipalities which participated in the JAGES 2013 survey, there were 13 municipalities where researchers declared that they were actively involved with and supported local health sector staff to utilize community assessment data and to collaborate with various organizations potentially contributing to health promotion for older adults. We designated these 13 specified municipalities as the intervention group, and the other 12 municipalities as the control group. The primary criterion for the intervention is that researchers provide the support for municipality health sector staff members to utilize the community assessment data tool (JAGES-HEART) and promote intersectoral collaboration, aiming to develop health-promoting social activities in the community. Although the backgrounds of the municipality health sector staff members working with JAGES varied, they were mostly public health nurses and administrative officers belonging to the municipality departments geared toward long-term care prevention, toward managing public long-term care insurance, or toward both.

In some municipalities, staff members from the Community-based Integrated Care Support Centers also participated. In each municipality, these municipality staff members were in charge of utilizing the data and other resources based on JAGES (e.g., JAGES study meetings, consultations with JAGES researchers, and peer counseling opportunities among participating municipality members) in order to take concrete actions for local purposes. For example, they utilized the results of epidemiologic evidence based on JAGES and “the community diagnosis sheet” based on JAGES-HEART in the community care meeting—the intersectoral meeting to which municipality officials from a wide range of sectors were invited—to discuss strategies for creating effective intersectoral policies to improve the health and well-being of local older people. In some municipalities, they utilized these data in the joint meetings with local representatives and the “Council for Social Welfare (*shakaifukushikyougikai*)” aiming to develop collaborative actions for improving daily lives in the community.

The backgrounds of the researchers participating in JAGES also varied, including such fields as gerontology, rehabilitation, dentistry, social welfare, social epidemiology, and public health, with various qualifications (e.g., physician, dentist, public health nurses, social welfare workers, and pharmacists). These researchers individually supported municipality staff members both in utilizing JAGES-HEART as a common tool and in facilitating meetings and workshops in which multiple sectors of the municipality and other local stakeholders discussed measures for improving older residents' health and well-being. These researchers have also provided information on relevant best practice and coached municipality staff members on decision making and promoting health and long-term care measures. Most of the interventions aimed to expedite municipalities' community-based prevention efforts against functional decline, as recommended by the central government: launching community places-to-go, or *kayoinoba* in Japanese (e.g., recreational salons), and increasing regular participation in those salons and in other existing formal/informal social activities. Researchers also provided information on successful examples of these activities in other municipalities and identified private companies interested in joining the community actions. The specific intervention approaches adopted during the collaboration between researchers and municipality staff members depended on the municipalities’ individual contexts and characteristics, and the health-related issues to be prioritized (see Suppl. 1). The details of the precise process and the specific interventions have been described elsewhere as a case study (Kondo and Rosenberg, 2018).

### 2.3 Measurements

#### 2.3.1 Social activities among residents

In both the JAGES 2013 and JAGES 2016 questionnaires, we asked about attendance frequency for eight different types of activities: sports group or club, leisure activity group, volunteer group, senior citizens’ club, neighborhood association, study or cultural group, health-building activities, and skill-teaching activities. We defined “group participation” as participating in any of the eight activities more than once a month, following a previous study based on JAGES (M. M. Saito et al., 2017).

In addition, the frequency of going out is another important behavioral aspect of social activity for older adults’ health independent of group participation, which can be a proxy for interactions with society/the neighborhood and physical activities. Previous studies have shown that a low frequency of going out is an important predictive factor for the incidence of physical or cognitive disability, rapid functional decline, and premature mortality among older people (Fujita et al., 2006; Saito et al., 2019). Thus, we also examined the frequency of going out as a proxy for the social activity level. We defined “infrequent going out” as going out an average of less than once a week (those responding “One to three times a month,” “Several times a year,” or “Rarely” to the single question “How often do you go out (including to the field or immediate neighborhood, for shopping, to the hospital, etc.”), in line with the definition in previous reports (Yasumura, 2003; Koyama et al., 2016) and of the Japanese Ministry of Health, Labour and Welfare (Ministry of Health, Labour and Welfare, 2009).

### 2.4 Covariates

Demographic variables included age, marital status (having a spouse or not), and living alone or not. We divided age into five categories; 65–69, 70–74, 75–79, 80–84, and 85 years and older. Socioeconomic status included equivalized annual household income, and educational attainment (less than 10 years or not). We used these cut-off points to ensure comparability with previous studies. We divided equivalized annual household income into tertiles in order to maximize statistical power. Physical or mental status included comorbidities, depressive symptoms, and declining instrumental activities of daily living (IADL). We assessed comorbidities as having any chronic diseases or disabilities. Our assessment of depressive symptoms was based on the Japanese short version of the Geriatric Depression Scale (GDS-15) developed for self-report surveys (Yesavage and Sheikh, 1986; Niino et al., 1991). We used the universally-accepted cut-off score of 4/5 (based on validation studies (Nyunt et al., 2009)) for indicating depressive tendency. We assessed IADL using the Tokyo Metropolitan Institute of Gerontology Index of Competence (TMIG-IC) (Koyano et al., 1991). We defined declining IADL as two or more ‘yes’ responses to the five TMIG-IC sub-items (T. T. Saito et al., 2017).

### 2.5 Statistical analysis

We conducted difference-in-differences (DID) estimation for comparing the changes in the prevalence and number of older residents’ participations in local activities between the intervention and control groups. We performed multilevel Poisson regression analyses to predict social participation and infrequent levels of going out; we also performed multilevel linear regression analyses to predict the number of participation activities for each group and year. We stratified the analysis by gender and adjusted for the covariates given above.

We did not randomly allocate the intervention and control groups, which could have led to selection bias. Using a logistic regression model applied to aggregated municipality-level data (N = 25), we calculated the propensity score of municipalities’ being selected for the intervention group, using the municipality-level variables potentially associated with the tendency to receive support from researchers: proportion of the population aged 65 or older, residential population density, proportion and incidence of receiving benefits from public long-term care insurance, standardized mortality ratio (SMR) of those aged 65 or older, financial power index, number of community places-to-go (such as recreational salons) operated by residents (per 10,000 residents aged 65 or older), duration of participating in JAGES, proportion of health professionals in the local health sectors, and maximum length of service of the local health sector staff members. We extracted these data from external data sources: the database of the Statistics Bureau in Japan, public LTCI operation status reports, annual hygiene and health statistics reports for each municipality, local finance surveys, surveys of the implementation status of long-term care prevention, and surveys of local health sector staff (Statistics Bureau, Ministry of Internal Affairs and Communications., 2013).

After examining standardized differences for comparing the baseline balance in the above variables between the intervention and control groups, we incorporated the inverse of the propensity score in the weighted multilevel Poisson regression analyses. We adopted this approach combining DID estimation and Inverse Probability of Treatment Weighting (IPTW), using propensity scores to make the observational data similar to an experimental study; this study can therefore be regarded as a quasi-experimental study (Geldsetzer and Fawzi, 2017).

We performed sensitivity analyses using the frequency of meetings between JAGES researchers and local health sector staff members (and other sectors or organizations) as the variable representing the magnitude of the intervention instead of the original intervention and control groups. In doing this, we assumed that the more frequently those meetings were held, the more action was being taken in terms of community organizing through collaboration between the researchers and local health sector staff members. This was because after 2013 there were some municipalities where collaboration did not proceed as expected or where some new collaborative approaches by researchers and local health sector staff members had begun, even though they were originally allocated as control groups at baseline. Based on the consultation frequency results for researchers and municipality staff members, we divided the frequency into three categories: more than twice per year (as representing systematic and continuous intervention through planning and conducting projects utilizing community diagnosis data), once or twice per year (likely to provide one-sided feedback on the community diagnosis data from researchers), and less than once a year (no consultation or feedback to municipalities). We consulted researchers to decide cut-off points for the content validity. In the sensitivity analyses, because of the difficulty in calculating the score due to strong correlations between the variables we used for the primary analysis, we simply adjusted for theoretically important municipality-level covariates instead of utilizing the propensity score. The adjusted variables included the proportion of those aged 65 or more, population density, incidence of receiving the LTCI benefit, SMR of those aged 65 or more, and number of places-to-go operated by residents, to avoid multicollinearity. We allowed for missing covariate values by assigning dummy variables for each missing category. We used Stata 14.1 for these statistical analyses (Stata Corp., Texas, USA).

### 2.6 Ethics approval

The JAGES survey protocol was approved by the Nihon Fukushi University ethics committee on research into human subjects (approval No. 13–14), Chiba University (approval No. 2493), and the National Centre for Geriatrics and Gerontology (approval No. 992). Data utilization for this study was approved by the University of Tokyo Faculty of Medicine ethics committee (approval No. 10555).

## 3 Results

**Fig. 1:**
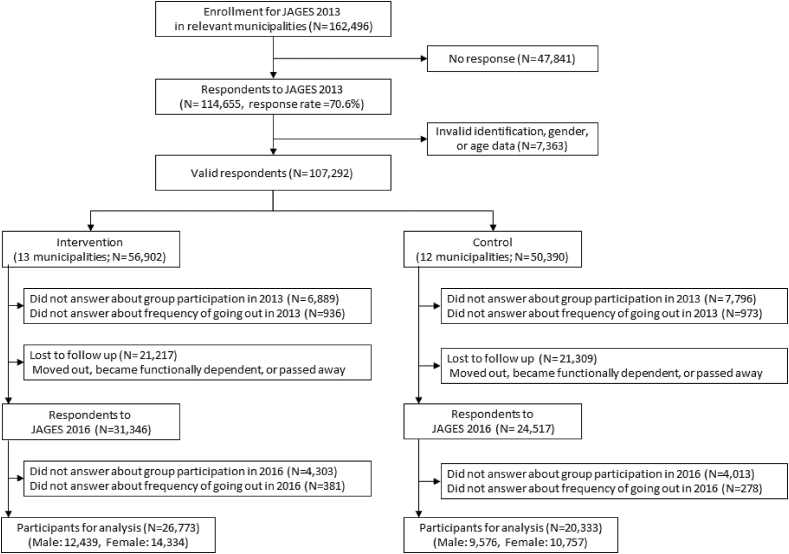
Flowchart of participants.

We analyzed data from 47,106 individuals (12,439 men and 14,334 women in the intervention group, 9576 men and 10,757 women in the control group) after excluding those with missing responses to group participation (n = 16,631) and frequency of going out (n = 1712) (Fig. 1, Table 1, Suppl. 2). After IPTW, all the characteristics of the municipalities were well-balanced between the intervention and control groups: standardized variable differences were less than 0.1 and c statistics 0.762 (Table 2). Descriptive statistics showed that group participation and infrequent going out scores for both men and women had increased in the three years.

**Table 1:** Baseline characteristics of the participants.

	Men				Women							
	Intervention (n = 12,439)		Control (n = 9576)		Intervention (n = 14,334)				Control (n = 10,757)			
	n	(%)	n	(%)	n		(%)		n		(%)	
Group participation in 2013	4706	(49.1)	6306	(50.7)	8688		(60.6)		6253		(58.1)	
Group participation in 2016	5303	(55.4)	7451	(59.9)	8678		(60.5)		6084		(56.6)	
Going out <1/week (infrequent going out) in 2013	358	(2.9)	219	(2.3)	383		(2.7)		247		(2.3)	
Going out <1/week (infrequent going out) in 2016	495	(4.0)	328	(3.4)	496		(3.5)		401		(3.7)	
Age												
65-69	3721	(29.9)	3401	(35.5)		4301		(30.0)		3540		(32.9)
70-74	4158	(33.4)	3040	(31.8)		4778		(33.3)		3440		(32.0)
75-79	2747	(22.1)	1891	(19.8)		3116		(21.7)		2218		(20.6)
80-84	1349	(10.8)	940	(9.8)		1587		(11.1)		1137		(10.6)
85-	464	(3.7)	304	(3.2)		552		(3.9)		422		(3.9)
Equivalent household income < 2 million yen	4996	(40.2)	3915	(40.9)		5795		(40.4)		4258		(39.6)
Education <10 years	3804	(30.6)	3911	(40.8)		5331		(37.2)		5373		(50.0)
Living alone	1126	(9.1)	602	(6.3)		2577		(18.0)		1471		(13.7)
No spouse	1639	(13.2)	1086	(11.3)		5159		(36.0)		3721		(34.6)
Having any comorbidities^a^	8969	(72.1)	6888	(71.9)		10,246		(71.5)		7837		(72.9)
Declining IADL^b^	704	(5.7)	773	(8.1)		393		(2.7)		425		(4.0)
Having depressive symptoms	2910	(23.4)	2344	(24.5)		3204		(22.4)		2475		(23.0)

a comorbidities = cancer, stroke, heart disease, hypertension, diabetes mellitus, dyslipidemia, respiratory disease, gastrointestinal disease or liver disease, kidney or prostate gland disease, musculoskeletal disease, traumatic injury, blood or immune system disease, psychiatric disease, dementia, Parkinson's disease, visual impairment, hearing impairment, and others.

b IADL= Instrumental Activities of Daily Living.

**Table 2:** Baseline characteristics of the municipalities.

	Intervention (n = 13)		Control (n = 12)		Standardized Difference	
	mean [SD]				Raw	Weighted
**Demographic**						
Proportion of aged ≥65, %	24.6	[5.0]	25.7	[8.3]	−0.24	−0.05
Proportion of older people using LTCI^a^, %	16.7	[2.7]	15.6	[1.5]	−0.05	−0.02
Incidence of certified LTCI^a^, %	4.6	[0.6]	5.2	[3.9]	−0.06	−0.03
Standardized Mortality Ratio (aged ≥65)	0.98	[0.07]	1.05	[0.08]	−0.607	−0.086
Financial Capability Index	0.7	[0.4]	0.7	[0.3]	0.254	0.076
City Index^b^	1.6	[0.8]	1.7	[0.5]	0.3	0.03
Number of community salons (/10,000 aged ≥65)	16.7	[31.1]	13.8	[19.6]	0.09	−0.09
Years since participating in JAGES	6.1	[4.0]	6.3	[3.8]	−0.22	−0.02
**Characteristics of municipality staff**						
Proportion of office workers	0.3	[0.3]	0.3	[0.3]	−0.03	0.02
Longest years in service	7.7	[6.8]	8.0	[5.3]	0.1	−0.03

SD = standard deviation.

a LTCI = Long-Term Care Insurance.

b City Index = Categories of residential population density (1: <1000/km^2^, 2: 1000–4000/km^2^, 3: >4000/km^2^).

**Fig. 2:**
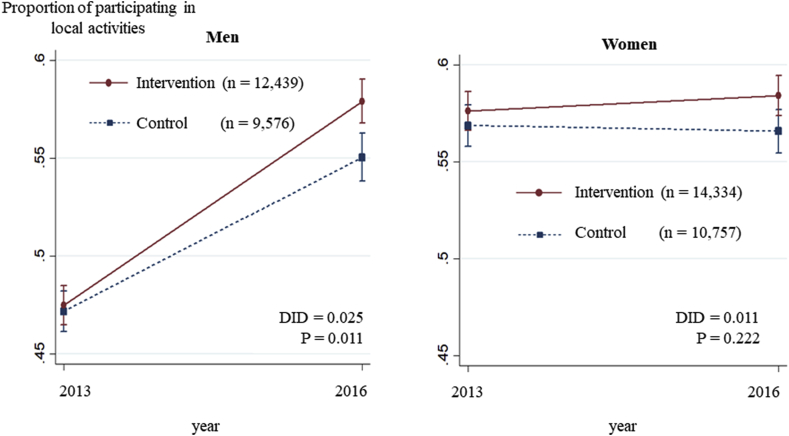
Changes in the proportion participating in local activities over three years: Results of Difference-in-Differences analysis.

**Fig. 3:**
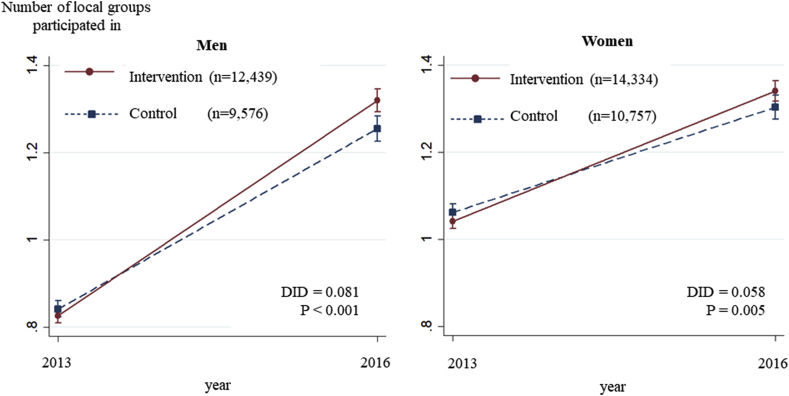
Changes in the number of local groups participated in over three years: Results of Difference-in-Differences analysis.

Among men, the estimated prevalence of group participation at baseline was 47.5% (95% confidence interval [CI]: 46.5%, 48.5%) in the intervention group and 47.2% (95% CI: 46.1%, 48.2%) in the control group. Three years later, group participation rose to 57.9% (95% CI: 56.8%, 59.0%) in the intervention group and 55.0% (95% CI: 53.8%, 56.3%) in the control group. The IPTW-DID of the intervention group against the control group was +2.5% (P = 0.011) (Table 3, Fig. 2, Suppl. 3). The number of participations in local activities also significantly rose from 0.83 (95% CI: 0.81, 0.84) to 1.32 (95% CI: 1.29, 1.35) in the intervention group (IPTW-DID = +0.08, P < 0.001) (Table 3, Fig. 3). Of the eight types of local activities, the DID tended to be especially high for leisure activity clubs and neighborhood associations. Among women, on the other hand, there were no significant changes in the predicted prevalence of group participation between the two groups across the three year period (Table 3, Fig. 2, Suppl. 3).

**Table 3:** Changes in infrequent going out status and in the proportion and number of groups participated in: results of difference-in-differences estimation.

	Intervention		Control		Difference-in-Differences	
	2013	2016	2013	2016	Predicted value	*P*
	Predicted value [95%CI*]	Predicted value [95%CI*]	Predicted value [95%CI*]	Predicted value [95%CI*]		
Infrequent going out						
Men	0.040 [0.037, 0.043]	0.050 [0.044, 0.055]	0.036 [0.033, 0.039]	0.044 [0.039, 0.049]	0.002	0.816
Women	0.041 [0.038, 0.044]	0.049 [0.044, 0.053]	0.036 [0.033, 0.039]	0.045 [0.042, 0.049]	−0.002	0.344
Groups Participation						
Men	0.475 [0.465, 0.485]	0.579 [0.568, 0.590]	0.472 [0.461, 0.482]	0.550 [0.538, 0.563]	0.025	0.011
Women	0.576 [0.566, 0.586]	0.584 [0.574, 0.595]	0.569 [0.558, 0.579]	0.566 [0.554, 0.577]	0.011	0.222
Number of activities						
Men	0.826 [0.810, 0.843]	1.320 [1.294, 1.345]	0.843 [0.824, 0.861]	1.255 [1.226, 1.284]	0.081	<0.001
Women	1.042 [1.025, 1.059]	1.341 [1.317, 1.364]	1.062 [1.043, 1.081]	1.303 [1.276, 1.330]	0.058	0.005
Proportion of those participating in specific activities (only men)						
Leisure activity club	0.275 [0.268, 0.282]	0.396 [0.385, 0.407]	0.274 [0.267, 0.282]	0.375 [0.363, 0.388]	0.020	0.027
Neighborhood association	0.123 [0.117, 0.129]	0.109 [0.102, 0.115]	0.113 [0.105, 0.120]	0.081 [0.073, 0.088]	0.017	<0.001
Volunteer group	0.125 [0.118, 0.131]	0.164 [0.157, 0.172]	0.118 [0.111, 0.124]	0.154 [0.147, 0.162]	0.003	0.933
Sports club or group	0.234 [0.226, 0.242]	0.309 [0.299, 0.319]	0.209 [0.199, 0.219]	0.287 [0.275, 0.298]	−0.002	0.246
Cultural group	0.064 [0.060, 0.067]	0.108 [0.102, 0.115]	0.053 [0.050, 0.057]	0.083 [0.076, 0.089]	0.015	0.125
Senior citizens' club	0.070 [0.065, 0.075]	0.090 [0.084, 0.097]	0.096 [0.087, 0.105]	0.118 [0.108, 0.128]	−0.002	0.429
Health-promoting activities	0.053 [0.049, 0.057]	0.090 [0.084, 0.096]	0.052 [0.049, 0.056]	0.091 [0.085, 0.098]	−0.002	0.696
Activities entailing passing on experience to others	0.056 [0.053, 0.060]	0.070 [0.065, 0.075]	0.058 [0.055, 0.062]	0.071 [0.066, 0.076]	0.001	0.683

The results of the sensitivity analysis using the researchers/municipality staff meeting frequency as representing the levels of intervention showed similar results. Those who lived in municipalities where the researchers/municipality staff meetings were held more than twice per year showed statistically significant increased participation in local activities, compared to those residents in municipalities where meetings were held less than once per year (P = 0.005) (Suppl. 4).

**Fig. 4:**
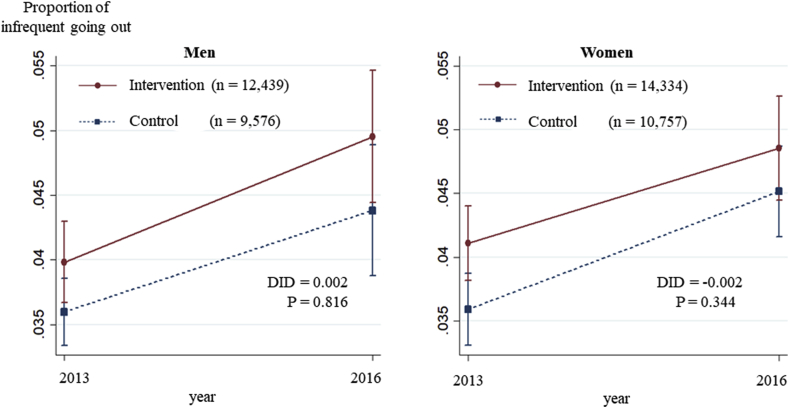
Changes in the proportion of infrequent going out over three years: Results of Difference-in-Differences analysis.

Among men, the predicted prevalence of infrequent going out was 4.0% (95% CI: 3.7%, 4.3%) in the intervention group and 3.6% (95% CI: 3.3%, 3.9%) in the control at baseline and 5.0% (95% CI: 4.4%, 5.5%) in the intervention group and 4.4% (95% CI: 3.9%, 4.9%) in the control at follow-up, with no statistically significant change between the two groups (DID = 0.2%, P = 0.816) (Table 3). On the other hand, in our sensitivity analysis, older men living in the municipalities where researchers/municipality staff meetings were held most frequently tended to have lower levels of infrequent going out than male residents of municipalities where meetings were held less frequently (P = 0.039). Among women, in the intervention group the predicted prevalence of infrequent going out changed from 4.1% (95% CI: 3.8%, 4.4%) to 4.9% (95% CI: 4.4%, 5.3%), whereas in the control group it changed from 3.6% (95% CI: 4.2%, 4.9%) to 4.5% (95% CI: 4.2%, 4.9%) during the three years, indicating no statistically significant differences (DID = -0.2%, P = 0.316) (Table 3, Fig. 4, Suppl. 3). The results were also similar when we used the frequency of meetings between researchers and health sector staff instead of the intervention (Suppl. 4).

## 4 Discussion

Overall, the prevalence of individual participation in social activities increased during the three years as did the number of groups individuals participated in. In municipalities where researchers empowered local health sector staff members, the increase in older male resident involvement in social activities was greater than in municipalities where only community diagnosis data were provided. The community organizing intervention was observed to be similarly effective in both the analysis using the originally assigned intervention and control group, and in the analysis using substantial collaboration between the researchers and local health sector staff members. However, the frequency of going out did not show a clear association with the intervention. The potential effects of the intervention for social activity participation among men were especially strong for participation in leisure activity groups and neighborhood associations. Women showed similar tendencies to men, but the association was statistically less clear.

The overall increase in participation in social activities is consistent with other reports in Japan, suggesting the similarity of our cohort to the general older population nationwide in Japan (Ministry of Health, Labour and Welfare, 2013). The results of our study on intervention effects are also similar to recent research in Japan. Shinkai et al. carried out a community intervention program including community empowerment directed at older residents at health check-up opportunities and showed an improvement in the functional health of older adults and decreased use of long-term care services (Shinkai et al., 2016). However, this was a single-arm trial without control groups and researchers approached residents directly. With our quasi-experimental study, we provide novel and stronger support for the idea that empowering municipality staff members, rather than older residents, is effective in improving participation levels in social activities among older male residents.

We consider two potential reasons for the increased participation in group activities only being statistically significant among men. First, our data indicated that, compared to women, there were more men who intended to participate in local activities at baseline, suggesting that men responded to the intervention more than women. Second, the statistically non-significant impact of the intervention on women may be due to ceiling effects or already high participation at baseline in both intervention and control municipalities. In other words, men of this generation who used to spend less time in their community than women when younger might benefit more from the community intervention. In many countries, persuading older men to participate in social activities has been a big challenge. Our findings suggest that community organizing interventions may be effective in addressing the challenge, making municipality staff members capable of identifying the challenges and opportunities related to the social inactivity of male residents. For example, the intervention may enable staff members to invite male residents to volunteer for or become steering committee members of neighborhood associations. Recent studies have suggested that targeted interventions directed at male residents for leisure or intellectual activities were successful (Okubo, 2005; Saito et al., 2015). This accords with our findings, showing that the intervention had a stronger impact on men in terms of leisure activity group participation.

On the other hand, our community intervention was not effective in reducing the prevalence of infrequent going out among both genders. There are several reasons that may explain this: given the extent to which this behavior was already established at baseline, our intervention may have been too weak to induce behavior change. It is also possible that we need to re-examine the validity and quality of our intervention or standardize the interventions (i.e., ways of making consultations to municipalities) more effectively. Finally, more strategic and comprehensive interventions might be required (e.g., the utilization of social marketing and involving local residents and organizations (Kamada et al., 2018)), improving local transportation, and making more green space available.

A number of study limitations need to be acknowledged. First, we used two time-point datasets. Thus, the parallel trend assumption between the empowerment group and the control group, which is a prerequisite for DID estimation, was not fully assured (Angrist and Pischke, 2008). However, the baseline proportions of social participation and infrequent going out were similar between groups, supporting the contention that baseline trends or characteristics were not significantly different between the two groups. Second, the effects shown might not be purely due to new interventions. There were pre-existing close collaborations between researchers and local staff members in several municipalities before 2013. Although our IPTW estimator addressed this potential selection bias in technical ways, it may not be perfect. Nonetheless, the well-balanced municipality characteristics between intervention and control municipalities support the contention that any potential bias would have been successfully addressed, either fully or to some extent. Third, the staff members in the municipalities participating in JAGES that were categorized as the control group might have been highly motivated and implemented various measures on their own accord, or may have received support from research organizations outside of JAGES. This would potentially cause an underestimation of the effect size we observed and could be a reason why we did not observe significant differences between the two groups in some analyses. Fourth, there must have been several steps between the intervention and impact. For example, the collaboration between researchers and local health sector staff members for utilizing community assessment data and building intersectoral partnerships may develop the municipality staff members' skills in sharing problems and setting goals with various sectors and institutions. Such efforts would smooth assessment and planning for health-promoting activities targeting social environments and involving older residents. Further research is needed to clarify this process and to evaluate the effectiveness of each step, just as it is also needed to evaluate why the interventions did not reduce the prevalence of infrequent going out among both genders, as noted previously. Fifth, we could not consider the precise frequency of participation in social activities or of going out owing to the questionnaire design. It might be necessary in future research to understand each individual's activity level as a whole and examine the dose-response effect. Finally, unobserved time-variant confounding might affect the results.

## 5 Conclusions

Collaborative partnerships between public health researchers and municipality staff members in quantitative community assessments and building intersectoral partnerships might be effective in increasing social participation among older male residents. Our study suggests the importance of establishing collaboration systems for local government in the health promotion of older adults. In the present study, researchers took the role of providing support. However, given that mobilizing public health researchers to carry out routine local activities nationwide is not realistic, the actual empowering support should utilize other resources from the public and private sectors. In Japan, the national government has started a new program of providing standardized support for municipalities in establishing local networks to provide community-integrated care for older adults by prefectural governments (Ministry of Health, Labour and Welfare, 2018). In terms of further research, given the short observational period in this study, the long-term effectiveness of such new interventions should be examined.
