# Supplementary material for: Effectiveness of community organizing interventions on social activities among older residents in Japan: A JAGES quasi-experimental study
Source: Soc Sci Med. 2019 Nov;240:112527. doi: 10.1016/j.socscimed.2019.112527 (PMC6880785; doi:10.1016/j.socscimed.2019.112527)

| Supplementary 1. List of intervention group municipalities | | |
| --- | --- | --- |
| Name of municipalities | Characteristics | Specific cooperative actions between municipalities and researchers |
| Taisetsu wide area union (includes Biei, Higashikawa, and Higashikagura towns) | Cold and snowy area, rural districts | Analyses are in progress for detailed additional data such as physical activity and cognitive function, eating habits and nutritional intake. |
|  |  |  |
| Iwanuma city | Disaster area, city | The city and research team work closely with each other on interventions related to prevention of functional disability in disaster recovery. |
| Kashiwa city | City | Advanced projects such as community-based integrated care systems are underway. |
| Yokohama city | Big city | Primary prevention-type activities are in progress. |
| Tokai city | City | The city is building an interaction base jointly with the local community association. Investigation of the health effects on older adults by researchers and the city is ongoing. |
| Toyohashi city | City | The city is widely running projects with researchers’ support to maintain a watch over older people who live alone and to develop residents' voluntary management salons for the prevention of functional decline. |
| Nagoya city | Big city with housing complexes | The researchers and local health sector staff members set up and opened salons for older people at housing complexes with growing older population. |
| Obu city | City | Local health sector staff members and researchers have collaborated and established local cooperation medical care systems. |
| Tahara city | Rural districts | The municipality promotes intersectoral action in line with the "healthy city declaration" through continuous meetings with researchers and local stakeholders. |
| Taketoyo town* | Mix of city area and rural districts | Researchers and local staff members strategically and successfully developed salons for older people based on a rigorous intervention research plan. The salons aimed to create places for older residents to gather on foot and to manage programs by themselves. The effectiveness of participating in those salons has been shown by the researchers. |
| Minamichita town | Coastal area, rural district | The researchers are examining the health effects on older people of a salon business launched by municipality health sector staff members and the residents. |
| Kobe city* | Big city | The researchers developed a community diagnosis tool using JAGES data and supported local health sector staff members to prioritize an area for launching salons for older people. The city started a salon project by collaborating with local companies at the prioritized area, and the researchers examined the effectiveness. |
| Matsuura city | Coastal area, rural district | Local health sector staff members implemented efforts for older people living in a prioritized area utilizing community diagnosis data provided by researchers. |
|  |  |  |
| Mifune town | Mountainous area, rural district | The researchers promoted intersectoral collaboration at conferences held by local health sector staff members. |
| *The detailed processes are described as case studies in another study (Kondo and Rosenberg, 2018). | | |

| Supplementary 2. Number and frequency of participations in local activities and times going out | | | | | | | | | | | |
| --- | --- | --- | --- | --- | --- | --- | --- | --- | --- | --- | --- |
|  | Men | | | | |  | Women | | | | |
|  | Intervention (n=12,439) | |  | Control (n=9,576) | |  | Intervention (n=14,334) | |  | Control (n=10,757) | |
|  | n | (%) |  | n | (%) |  |  | (%) |  | n | (%) |
| Number of participations in local activities in 2013 |  |  |  |  |  |  |  |  |  |  |  |
| 0 | 7,735 | (62.2) |  | 6,047 | (63.2) |  | 7,827 | (54.6) |  | 6,114 | (56.8) |
| 1 | 2,031 | (16.3) |  | 1,536 | (16.0) |  | 2,509 | (17.5) |  | 1,800 | (16.7) |
| 2 | 1,366 | (11.0) |  | 1,029 | (10.8) |  | 1,853 | (12.9) |  | 1,300 | (12.1) |
| 3 | 639 | (5.1) |  | 512 | (5.4) |  | 1,117 | (7.8) |  | 755 | (7.0) |
| 4-8 | 668 | (5.4) |  | 452 | (4.7) |  | 1,028 | (7.2) |  | 788 | (7.3) |
| Number of participations in local activities in 2016 |  |  |  |  |  |  |  |  |  |  |  |
| 0 | 4,988 | (40.1) |  | 4,273 | (44.6) |  | 5,656 | (39.5) |  | 4,673 | (43.4) |
| 1 | 2,472 | (19.9) |  | 1,804 | (18.8) |  | 2,895 | (20.2) |  | 1,833 | (17.0) |
| 2 | 2,329 | (18.7) |  | 1,628 | (17.0) |  | 2,675 | (18.7) |  | 1,833 | (17.0) |
| 3 | 1,306 | (10.5) |  | 962 | (10.1) |  | 1,530 | (10.7) |  | 1,043 | (9.7) |
| 4-8 | 1,344 | (10.8) |  | 911 | (9.5) |  | 1,578 | (11.0) |  | 1,093 | (10.2) |
| Frequency of participation in the activities with the most participation in 2013 |  |  |  |  |  |  |  |  |  |  |  |
| >4/week | 1,097 | (8.8) |  | 899 | (9.4) |  | 1,406 | (9.8) |  | 1,072 | (10.0) |
| 2-3/week | 1,735 | (14.0) |  | 1,132 | (11.8) |  | 2,753 | (19.2) |  | 1,946 | (18.1) |
| 1/week | 1,277 | (10.3) |  | 971 | (10.1) |  | 2,201 | (15.4) |  | 1,540 | (14.3) |
| 1-3/month | 2,197 | (17.7) |  | 1,704 | (17.8) |  | 2,328 | (16.2) |  | 1,695 | (15.8) |
| a few times per year | 2,355 | (18.9) |  | 2,197 | (22.9) |  | 1,820 | (12.7) |  | 1,548 | (14.4) |
| never | 3,778 | (30.4) |  | 2,673 | (27.9) |  | 3,826 | (26.7) |  | 2,956 | (27.5) |
| Frequency of participation activities with the most participation in 2016 |  |  |  |  |  |  |  |  |  |  |  |
| >4/week | 1,362 | (11.0) |  | 1,038 | (10.8) |  | 1,584 | (11.1) |  | 1,215 | (11.3) |
| 2-3/week | 2,182 | (17.5) |  | 1,577 | (16.5) |  | 2,643 | (18.4) |  | 1,747 | (16.2) |
| 1/week | 1,651 | (13.3) |  | 1,116 | (11.7) |  | 1,898 | (13.2) |  | 1,301 | (12.1) |
| 1-3/month | 2,256 | (18.1) |  | 1,572 | (16.4) |  | 2,553 | (17.8) |  | 1,821 | (16.9) |
| a few times per year | 1,609 | (12.9) |  | 1,479 | (15.4) |  | 1,856 | (13.0) |  | 1,620 | (15.1) |
| never | 3,379 | (27.2) |  | 2,794 | (29.2) |  | 3,800 | (26.5) |  | 3,053 | (28.4) |
| Frequency of going out in 2013 |  |  |  |  |  |  |  |  |  |  |  |
| >4/week | 9,605 | (77.2) |  | 7,752 | (81.0) |  | 10,662 | (74.4) |  | 8,363 | (77.7) |
| 2-3/week | 2,057 | (16.5) |  | 1,341 | (14.0) |  | 2,808 | (19.6) |  | 1,826 | (17.0) |
| 1/week | 419 | (3.4) |  | 264 | (2.8) |  | 481 | (3.4) |  | 321 | (3.0) |
| 1-3/month | 286 | (2.3) |  | 164 | (1.7) |  | 323 | (2.3) |  | 199 | (1.9) |
| a few times per year | 40 | (0.3) |  | 32 | (0.3) |  | 31 | (0.2) |  | 24 | (0.2) |
| never | 32 | (0.3) |  | 23 | (0.2) |  | 29 | (0.2) |  | 24 | (0.2) |
| Frequency of going out in 2016 |  |  |  |  |  |  |  |  |  |  |  |
| >4/week | 8,984 | (72.2) |  | 7,259 | (75.8) |  | 10,471 | (73.1) |  | 8,121 | (75.5) |
| 2-3/week | 2,536 | (20.4) |  | 1,659 | (17.3) |  | 2,860 | (20.0) |  | 1,857 | (17.3) |
| 1/week | 424 | (3.4) |  | 330 | (3.5) |  | 507 | (3.5) |  | 378 | (3.5) |
| 1-3/month | 406 | (3.3) |  | 246 | (2.6) |  | 412 | (2.9) |  | 308 | (2.9) |
| a few times per year | 48 | (0.4) |  | 42 | (0.4) |  | 50 | (0.4) |  | 47 | (0.4) |
| never | 41 | (0.3) |  | 40 | (0.4) |  | 34 | (0.2) |  | 46 | (0.4) |

| Supplementary 3. Prevalence ratio of group participation and infrequent going out by intervention and control group | | | | | | | | |
| --- | --- | --- | --- | --- | --- | --- | --- | --- |
|  | Group participation | | | |  | Infrequent going out | | |
|  | Men (n=22,015) |  | Women (n=25,091) | |  | Men (n=22,015) |  | Women (n=25,091) |
|  | PR (95% CI) |  | PR (95% CI) | |  | PR (95% CI) |  | PR (95% CI) |
| Fixed Effect |  |  |  | |  |  |  |  |
| Intercept | 0.52 [0.51, 0.54] |  | 0.63 [0.62, 0.65] | |  | 0.007 [0.005, 0.009] | | 0.006 [0.004, 0.008] |
| Year | 1.17 [1.14, 1.19] |  | 0.99 [0.97, 1.02] | |  | 1.26 [1.12, 1.42] |  | 1.31 [1.19, 1.44] |
| Intervention (ref. Control) | 1.01 [0.98, 1.04] |  | 1.01 [0.99, 1.04] | |  | 1.13 [1.01, 1.26] |  | 1.15 [1.03, 1.28] |
| Intervention × year | 1.04 [1.01, 1.08] |  | 1.02 [0.99, 1.05] | |  | 1.02 [0.85, 1.22] |  | 0.93 [0.82, 1.06] |
| Age (ref: 65-69) |  |  |  | |  |  |  |  |
| 70-74 | 1.11 [1.08, 1.13] |  | 1.08 [1.06, 1.10] | |  | 1.04 [0.92, 1.16] |  | 1.09 [0.97, 1.21] |
| 75-79 | 1.16 [1.13, 1.19] |  | 1.10 [1.08, 1.13] | |  | 1.27 [1.14, 1.41] |  | 1.38 [1.23, 1.55] |
| 80-84 | 1.12 [1.08, 1.16] |  | 1.09 [1.06, 1.12] | |  | 1.56 [1.35, 1.79] |  | 1.74 [1.53, 1.99] |
| 85- | 1.01 [0.96, 1.06] |  | 0.99 [0.94, 1.03] | |  | 2.05 [1.74, 2.40] |  | 2.43 [2.08, 2.83] |
| Education < 10 years (ref: ≥10 years) | 0.86 [0.84, 0.88] |  | 0.87 [0.85, 0.88] | |  | 1.14 [1.05, 1.24] |  | 1.02 [0.95, 1.10] |
| Tertiles of equivalent household income (ref: T3 (Highest income)) | | |  |  |  |  |  |  |
| T2 (Middle income) | 0.92 [0.90, 0.94] |  | 0.92 [0.91, 0.94] | |  | 1.26 [1.13, 1.40] |  | 1.26 [1.14, 1.39] |
| T1 (Lowest income) | 0.96 [0.94, 0.97] |  | 0.98 [0.96, 1.00] | |  | 1.03 [0.93, 1.16] |  | 0.97 [0.86, 1.10] |
| No spouse (ref: having spouse) | 0.99 [0.95, 1.03] |  | 1.07 [1.04, 1.09] | |  | 0.90 [0.75, 1.08] |  | 0.91 [0.83, 0.99] |
| Living alone (ref: living with somebody) | 0.94 [0.91, 0.97] |  | 0.98 [0.96, 1.00] | |  | 1.21 [1.06, 1.37] |  | 1.05 [0.97, 1.13] |
| Having depressive symptoms (ref: not depressive) | 0.81 [0.79, 0.82] |  | 0.82 [0.80, 0.83] | |  | 1.56 [1.43, 1.70] |  | 1.53 [1.41, 1.66] |
| Having any comorbidities (ref: no comorbidities) | 0.99 [0.97, 1.02] |  | 1.00 [0.98, 1.02] | |  | 1.27 [1.09, 1.47] |  | 1.34 [1.18, 1.51] |
| Declining IADL (ref: no decline) | 0.75 [0.72, 0.78] |  | 0.59 [0.56, 0.63] | |  | 3.33 [3.04, 3.64] |  | 4.40 [3.92, 4.93] |
| Random effect |  |  |  | |  |  |  |  |
| Variance of school district intercept | 0.002 [0.001, 0.004] |  | 0.002 [0.001, 0.003] | |  | 0.09 [0.05, 0.15] |  | 0.08 [0.05, 0.13] |
| Variance of individual intercept | 4.2E^-41^ [4.7E^-46^, 3.7E^-36^] |  | 3.2E^-39^ [2.4E^-42^, 4.1E^-38^] | |  | 0.77 [0.44, 1.34] |  | 0.71 [0.37, 1.34] |
| PR=Prevalence Ratio, CI=Confidence Interval, IADL= Instrumental Activities of Daily Living | | | | | | | | |

| Supplementary 4. Prevalence ratio of group participation and infrequent going out by frequency of meeting between researchers and health sector staff | | | | | | | | | | | | |  |  |
| --- | --- | --- | --- | --- | --- | --- | --- | --- | --- | --- | --- | --- | --- | --- |
|  | Group participation | | | | |  | | Infrequent going out | | | | |  |  |
|  | Men (n=22,015) |  | Women (n=25,091) | | |  | Men (n=22,015) | | | |  | Women (n=25,091) | | |
|  | PR (95% CI) |  | PR (95% CI) | | |  | PR (95% CI) | | | |  | PR (95% CI) | | |
| Fixed Effect |  |  |  | | |  |  | | | |  |  | | |
| Intercept | 0.732 [0.591, 0.906] |  | 0.004 [0.002, 0.009] | | |  | 0.999 [0.978, 1.022] | | | |  | 0.936 [0.767, 1.142] | | |
| Year | 1.18 [1.15, 1.20] |  | 1.22 [1.12, 1.33] | | |  | 1.31 [1.18, 1.46] | | | |  | 1.00 [0.98, 1.02] | | |
| Frequency of meeting（ref: <1/year) |  |  |  | | |  |  | | | |  |  | | |
| 1-2/year | 1.00 [0.96, 1.04] |  | 1.05 [0.92, 1.20] | | |  | 1.05 [0.91, 1.21] | | | |  | 1.03 [0.99, 1.07] | | |
| ≥3/year | 0.92 [0.90, 0.95] |  | 1.01 [0.90, 1.14] | | |  | 1.01 [0.88, 1.15] | | | |  | 0.96 [0.93, 0.99] | | |
| Year×Frequency of meeting |  |  |  | | |  |  | | | |  |  | | |
| 1-2/year | 1.00 [0.96, 1.04] |  | 1.02 [0.87, 1.19] | | |  | 1.05 [0.87, 1.27] | | | |  | 1.01 [0.98, 1.05] | | |
| ≥3/year | 1.06 [1.02, 1.10] |  | 0.97 [0.83, 1.13] | | |  | 0.84 [0.71, 0.99] | | | |  | 1.02 [0.98, 1.06] | | |
| Age (ref: 65-69) |  |  |  | | |  |  | | | |  |  | | |
| 70-74 | 1.11 [1.08, 1.13] |  | 1.08 [0.98, 1.19] | | |  | 1.06 [0.96, 1.18] | | | |  | 1.07 [1.05, 1.09] | | |
| 75-79 | 1.15 [1.13, 1.18] |  | 1.39 [1.26, 1.54] | | |  | 1.26 [1.13, 1.40] | | | |  | 1.10 [1.08, 1.12] | | |
| 80-84 | 1.12 [1.09, 1.15] |  | 1.68 [1.49, 1.90] | | |  | 1.52 [1.34, 1.72] | | | |  | 1.08 [1.05, 1.10] | | |
| 85- | 1.01 [0.97, 1.05] |  | 2.27 [2.00, 2.59] | | |  | 2.04 [1.77, 2.36] | | | |  | 0.97 [0.94, 1.01] | | |
| Education ≤ 9 years (ref: >9 years) | 0.87 [0.86, 0.89] |  | 0.95 [0.89, 1.02] | | |  | 1.09 [1.01, 1.17] | | | |  | 0.87 [0.86, 0.88] | | |
| Tertiles of equivalent household income (ref: T3 (Highest income)) | |  | |  |  |  | | |  |  | | |  |  |
| T2 (Middle income) | 0.92 [0.90, 0.94] |  | 1.19 [1.09, 1.30] | | |  | 1.23 [1.12, 1.36] | | | |  | 0.92 [0.9, 0.94] | |  |
| T1 (Lowest income) | 0.96 [0.94, 0.98] |  | 0.95 [0.85, 1.06] | | |  | 1.03 [0.93, 1.15] | | | |  | 0.98 [0.97, 1.00] | |  |
| No spouse (ref: having spouse) | 0.98 [0.94, 1.02] |  | 0.91 [0.83, 0.99] | | |  | 0.91 [0.78, 1.08] | | | |  | 1.07 [1.05, 1.09] | |  |
| Living alone (ref: living with somebody) | 0.94 [0.91, 0.97] |  | 1.06 [0.98, 1.14] | | |  | 1.21 [1.08, 1.36] | | | |  | 0.98 [0.96, 1.00] | |  |
| Having depressive symptoms (ref: not depressive) | 0.80 [0.79, 0.82] |  | 1.49 [1.40, 1.60] | | |  | 1.53 [1.41, 1.66] | | | |  | 0.83 [0.81, 0.84] | |  |
| Having any comorbidities (ref: no comorbidities) | 0.99 [0.97, 1.01] |  | 1.33 [1.19, 1.48] | | |  | 1.24 [1.10, 1.40] | | | |  | 1.00 [0.99, 1.02] | |  |
| Declining IADL (ref: no decline) | 0.76 [0.73, 0.79] |  | 4.19 [3.79, 4.63] | | |  | 3.19 [2.96, 3.45] | | | |  | 0.59 [0.55, 0.62] | |  |
| Proportions of aged ≥65, % | 0.996 [0.991, 1.001] |  | 1.047 [1.035, 1.060] | | |  | 1.049 [1.034, 1.063] | | | |  | 0.994 [0.990, 0.998] | |  |
| Incidence of certified LTCI, % | 0.998 [0.992, 1.004] |  | 0.988 [0.980, 0.996] | | |  | 0.978 [0.966, 0.991] | | | |  | 1.002 [0.999, 1.005] | |  |
| Standardized Mortality Ratio (aged ≥65) | 0.799 [0.648, 0.986] |  | 0.905 [0.480, 1.707] | | |  | 0.917 [0.405, 2.078] | | | |  | 0.749 [0.637, 0.880] | |  |
| Number of community salons (/10,000 aged ≥65） | 1.001 [1.000, 1.001] |  | 1.001 [0.999, 1.004] | | |  | 1.004 [1.001, 1.006] | | | |  | 1.001 [1.000, 1.002] | |  |
| Residential population density (1000/km^2^) | 1.009 [1.006, 1.012] |  | 0.990 [0.972, 1.007] | | |  | 0.999 [0.978, 1.022] | | | |  | 1.011 [1.007, 1.014] | |  |
| Random effect |  |  |  | | |  |  | | | |  |  | |  |
| Variance of school district intercept | 3.0E^-38^ [1.3E^-98^, 7.1E^+22^] | 0.032 [0.014, 0.07] | | |  | 0.049 [0.027, 0.089] | | |  | 0.013 [0.001, 0.133] | | |  |  |
| Variance of individual intercept | 9.2E^-37^ [4.9E^-37^, 1.7E^-36^] | 1.4E^-33^ [1.2E^-33^, 1.7E^-33^] | | | | 5.8E^-32^ [7.4E^-32^, 4.7E^-31^] | | | | 1.3E^-32^ [3.8E^-33^, 4.3E^-32^] | | |  |  |
| PR=Prevalence Ratio, CI=Confidence Interval, IADL=Instrumental Activities of Daily Living, LTCI=Long-Term Care Insurance | | | | | | | | | | | | | |  |


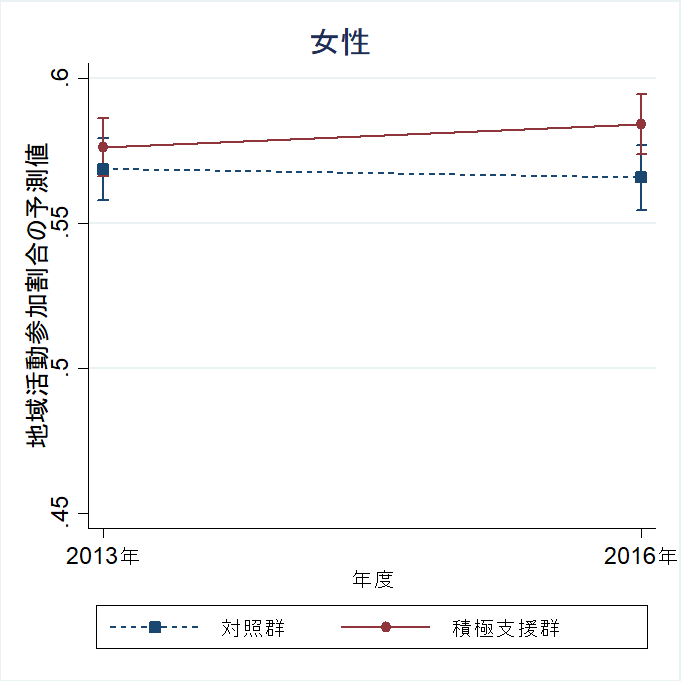

Supplement: Multimedia component 1 [file mmc1.docx]
